# Transactions of the Odontological Society of Great Britain

**Published:** 1879-11

**Authors:** 


### ARTICLE II.

[Continued from last Number.]

### *Transactions of the Odontological Society of Great Britain.*

The President then called upon Mr. Arthur Underwood to read his paper

### On the Functions of the Nerves of Taste.

*Mr. President and Gentlemen.*—My interest has been aroused concerning the subject upon which I have the honour to address you to-night by a paper upon the question forwarded to me some months ago from Dublin.

The author, Dr. Nixon, after giving the particulars of a case of double facial paralysis, enters somewhat fully into the more recent opinions of physiologists upon the nerve supply of taste; and, having read his remarks with great interest myself, I thought some *resume* of the kind might prove interesting to this Society. Cases very similar to Dr. Nixon's are being constantly brought forward in the medical papers, and the conclusions to which they point appear in a more forcible light when the evidence is grouped than when it is isolated.

For a long time the almost universally acknowledged view of physiologists was that the sense of taste was conveyed to the cerebrum by the agency of two nerves—the glossopharyngeal and the lingual branch of the 5th pair—the former presiding over taste at the root of the tongue, the latter at the tip and sides. This opinion was supported by the apparently conclusive evidence that section of either nerve produced loss of taste in the region it supplied.

The actual result of the experiment was true. The deductions of the experimenters, as is often the case, have been since shown to be mistaken as far as the lingual was concerned. Since the question first became a matter of dispute the controversy has led to many and various opinions being alternately entertained, and then, as evidence accumulated, abandoned. I do not think there is perfect unanimity upon the subject yet, but there is at least a growing inclination to adopt one view among a large section of physiologists.

I would premise that the title of the glossopharyngeal to supply the special sense to the root of the tongue never having been disputed I shall not allude to it, and my reference to the sense of taste in the future part of the paper will be understood to mean the sense in the antero-lateral portion of the tongue only.

During the discussion of the question certain important facts have been fully established.

1. Section of the lingual after the chorda tympani has joined it produces loss of common sensation and loss of taste.

2. Section of the lingual before the chorda tympani joins it produces loss of common sensation, but does not affect the sense of taste.

3. Section of the chordi tympani before it joins the lingual produces loss of taste, but does not affect common sensation.

Furthermore, the evidence of disease is that—

1. Complete paralysis of the 5th pair, including of course the lingual, affects sensation, but not taste. (*Vide* Dr. Althaus' case—*Trans. Med. Chi.* Vol. LII.)

2. Paralysis of the 7th pair (due to lesion in its interpetrosal course) affects the sense of taste, but not common sensation. (*Vide* Dr. McDonnell's case--*Trans. Med. Chi.*, Vol. LVIII.)

The evidence by which these facts have been established is so voluminous that it would be impossible to reproduce it here, but if any gentlemen would be interested to hear the particulars of the experiments in the cases of paralysis I shall be happy to quote some of them in my reply.

These facts point at once to one conclusion—that the theory that the lingual *per se* has any influence over the special sense of taste must be abandoned, it being clear that such influence is transmitted to it by the chorda tympani.

Whence, then, does the chorda tympani derive this power over a special sense?

That it has it before it leaves the 7th in the aqueduct of Sylvius is plain from the fact that lesion of that nerve in that situation, either by disease, as in Dr. McDonnell's and Dr. Nixon's case, or injury, as in the cases of Vizioli, Stick, or Lotzbeck, produces loss of taste.

It is equally evident that the 7th itself cannot communicate the power, for two reasons:

First, because the portia dura is a purely motor nerve, and could scarcely be accredited with a special sense.

Secondly, because central paralysis of the portio dura, or section of it nearer to its origin than the gangliform enlargement, does not affect the sense. (Austin Flint, Hughlings Jackson, Hermann.)

In the case of the 7th, as with the lingual, the chorda tympani is only a guest, and not an offspring.

The next step in tracing this influence back to its cerebral source was to discover by what channel the chorda tympani joined the 7th.

There are four routes by which it may do so.

1. *Via* the great superficial petrosal from Meckel's ganglion.

2. *Via* the lesser superficial petrosal from the otic ganglion.

3. *Via* the external superficial petrosal from the sympathetic plexus on the middle meningeal.

4. *Via* the "nervus anastomoticus" glossopharyngeal outside the stylomastoid foramen.

No. 3 may be dismissed at once, as being simply vasomotor from the sympathetic.

In cases 1 and 2, that is from Meckel's ganglion, or from the otic, we should again be referred to the 5th pair for the original source.

At this point I must allude to a series of experiments by Schiff, undertaken with a view of clearing up this point.

He divided the 2nd division of the 5th above Meckel's ganglion; then the branches going to Meckel's ganglion; then the great superficial petrosal nerve, and finally removed Meckel's ganglion altogether.

His conclusion was that some of the taste fibres at least left the cerebrum with the 5th pair, passing along the 2nd division to Meckel's ganglion, reaching the 7th from thence by the great superficial petrosal, and leaving it as chorda tympani.

In support of this view is the analogy of the horse, in which animal, as was shown by Professor Owen, the great superficial petrosal leaves the portio dura as chorda tympani without becoming incorporated with its fibres at all.

Yet this view has, I think, been shown to be incorrect, both by the experiments of man and of nature.

Vulpian and Prevost repeated Schiff's directions with different results, and showed that after ablation of Meckel's ganglion the sense of taste persisted. But, after all, dissections involving such extreme nicety are very liable to error. Again, it is difficult to be sure about the persistence or extent of the sense of taste in the lower animals, their power of communicating their impressions being necessarily limited; and valuable as experimental evidence is, evidence derived from the observation of the results of disease or accident upon the human subject is still more satisfactory and conclusive, not only because the dissections are more exact—the experiments, in fact, conducted with greater nicety, and with less implication of other nerves—but also because a human patient can give the observer a better account of his own sensations.

It is then to the results of disease that I now turn for further elucidation of the matter. The two cases I allude to are chosen from very many because of the very high authority upon which they rest, and because they are eminently typical and very conclusive.

In the 52nd volume of the *Med. Chi. Tr.*, Dr. Althaus quotes a case of complete loss of function of the whole of the 5th pair unaccompanied by any other lesion. The loss of common sensation over the front portion of the tongue was so complete that the organ was actually wounded by the teeth without the patient being conscious of the fact.

The sense of taste was quite unaffected, and this was demonstrated by a series of delicate and ingenious experiments, the details of which are given in the Transactions.

The second case is one cited by Dr. McDonnell, also in the *Med. Chi. Tr.* (Vol. LVIII.)

It is a case of paralysis of the 7th, or portio dura, due to disease of its interpetrosal portion. In this case, while common sensation over the front of the tongue was as keen as in the doctor himself or any of the surrounding students, the power of taste in that region was quite lost.

The paper by Dr. Nixon, of Dublin, to which I am indebted for much of the material of the present paper, contains the particulars of a similar case to Dr. McDonnell's.

These are types of numerous cases, all of which show that though the special sense undoubtedly leaves the portio dura by the chorda tympani, it does not reach it from Meckel's ganglion, or from any other part of the 5th pair.

Is there then any other source of influence to the facial besides Meckel's ganglion and the otic? I have already alluded to a communication from the glossopharyngeal reaching the facial outside the stylomastoid foramen, called the "nervus anastomoticus," but this is not the only communication the glossopharyngeal sends to the 7th.

The glossopharyngeal gives off a tympanic branch which communicates with both the greater and the lesser superficial petrosal nerves between their ganglia and their union with the facial. Here then is an influence reaching the facial by the petrosal nerves, which would obviously not be disturbed either by paralysis of the 5th pair or by the removal of Meckel's ganglion. Moreover, it is a significant fact that this influence is derived from a nerve (the glossopharyngeal) which has always been regarded as undoubtedly a special nerve of taste.

According to this view then the glossopharyngeal would preside over the whole sense of taste, both at the root and over the tip and sides of the tongue. And I must urge that it seems more in accordance with common sense to refer this taste-sense to the empire of one nerve and not two.

It is more in accordance with analogy, as such a phenomenon as a special sense depending on two nerves is unparelleled in nature. Sight, hearing, smell, each has its nerve specially adapted to convey its special impressions to the sensorium. They are not apparently in need of assistance from a motor or a sensory nerve to carry out their function. Why should it not be the same in the case of taste?

Anatomy shows us an unbroken line of communication between the glossopharyngeal and the tip and sides of the tongue. To recapitulate the chain, it runs from the glossopharyngeal by the tympanic branch to the petrosal nerves to the facial, leaves the facial as chorda tympani, joins the lingual, and so to the tip and sides of the tongue.

Experimental dissection and disease both point, as I have endeavored to show, to the fact that if this line of communication be interrupted the sense of taste over that region is lost; that if the chain of communication be left intact no other dissections or injuries affect the sense.

Analogy would suggest that there is likely to be only one nerve of taste,

The title of the glossopharyngeal to be considered a special nerve of taste has never been disputed.

From the due consideration of these facts, I myself can have no hesitation in arriving at this conclusion, as far as the light thrown upon the subject warrants any conclusion, that the glossopharyngeal is the only nerve of taste, and that the 2nd and 3rd divisions of the 5th pair have as little to do with this sense as the 1st division has to do with the sense of sight.

Of course there are many minor difficulties to be cleared up, and I do not doubt that in advancing a view that, although sanctioned by Herman, Dr. McDonnell, Dr. Althaus and many others, can scarcely be said to be universally accepted, I lay myself open to questions I may not be able to answer, and arguments I cannot demolish. I think there is a very strong case for the glossopharyngeal, which will also take much to demolish it.

[TO BE CONTINUED.]

